# Complex coacervation of anthocyanin-rich pigments from red cabbage (*Brassica oleracea*) with inulin, gum arabic and pea protein

**DOI:** 10.3389/fnut.2025.1523365

**Published:** 2025-03-13

**Authors:** Sandra Muñoz-Coyotecatl, Astrid Domínguez-Uscanga, Randy Ortiz-Castro, Greta H. Rosas-Saito, Gregorio Romero-De la Vega, Genaro Amador-Espejo, Diego A. Luna-Vital

**Affiliations:** ^1^Tecnológico de Monterrey, School of Engineering and Science, Monterrey, Mexico; ^2^Tecnológico de Monterrey, Institute for Obesity Research, School of Bioengineering and Science, Monterrey, Mexico; ^3^Instituto de Ecología, A.C., Red de Estudios Moleculares Avanzados, Edificio B, Xalapa, Veracruz, Mexico; ^4^Universidad Iberoamericana Puebla-IDIT, Boulevard del Niño Poblano, Reserva Territorial Atlixcáyotl, Puebla, Mexico; ^5^Centro de Investigación en Biotecnología Aplicada, Tepetitla, Tlaxcala, Mexico

**Keywords:** complex coacervation, anthocyanin, red cabbage, natural pigments, microencapsulation

## Abstract

Complex coacervation is a widely used method for bioactive compound microencapsulation. Red cabbage extract is a natural pigment that contains anthocyanins, which provide attractive and bright colors with no reported toxicity and associated healthy properties. These types of pigments have led to a deep interest in developing natural colorants to at least partially replace their synthetic counterparts in the food industry. The present study aimed to encapsulate red cabbage extract using a complex coacervation system comprising gum arabic:inulin (GA:In) and pea protein (PP) as wall materials. A total of four treatments were tested, maintaining a consistent pea protein concentration (1%), and the concentrations of GA:In (1 and 3%) and red cabbage extract (1 and 10%) were varied. The results showed high encapsulation efficiency values, with all treatments achieving encapsulation levels above 95%. The total monomeric anthocyanin concentration was 6.7 μg anthocyanin Eq. C3G/mg of flour, and to explore bioactivity of the extract, *α*-amylase inhibition was analyzed, with an inhibitory percentage of 22.48% at a concentration of 0.5 mg/mL. The solubility of the coacervates ranged from 70.12 to 75.84% in water, and their morphology revealed irregular and porous shapes. Fourier Transform Infrared Spectroscopy (FTIR) analysis confirmed the formation of the coacervate-encapsulation complex. Characteristic bands showed the presence of functional groups from the wall materials and the encapsulated anthocyanins. These findings showed that the use of GA, In and PP as wall materials in complex coacervation can develop natural colorants with improved stability and functionality.

## 1 Introduction

In the field of food science, there is a constant search for encapsulation technology solutions. One of the main goals is to improve the stability and effectiveness of bioactive compounds in preventing or treating health problems, which in turn has led to the development and study of various innovative methods and systems. One of the most promising approaches in this area is microencapsulation, a technique that allows for the protection and controlled release of active substances (1).

Anthocyanins are water-soluble pigments that belong to a group of natural compounds called flavonoids. Anthocyanins are glucosides of anthocyanidins. They are made up of an anthocyanidin molecule (aglycone) and a sugar connected to it by a *β*-glycosidic bond (2). These pigments play an important role in the color quality of flowers, cereals, berries, vegetables, and fruits that are red, purple, or blue in color (3). Various parts of plants, including flowers, stems, fruits, leaves, and roots, serve as the main source of anthocyanins (4). Anthocyanins provide attractive and bright colors with no reported toxicity, which has led to a deep interest in developing natural colorants to partially replace their synthetic counterparts for use in the food industry (5). Applications of natural pigments include wines, confectionery, juices, sweets, among other food matrices (6). Including anthocyanins as natural colorants in food products, not only contributes to improving the overall appearance, but it could add value due to their potential health benefits. For instance, they have been found to possess potential anti-diabetic and anti-inflammatory properties and contribute to the prevention of cardiovascular diseases and some types of cancer (7, 8). However, despite the studies conducted, the use of these pigments is limited due to their instability and sensitivity to environmental factors, such as light, temperature, pH, and humidity (9, 10). Microencapsulation is a promising technique for protecting anthocyanins from degradation and improving their stability and shelf life to increase their use in the food industry, consumption, and, therefore, their beneficial effect on health.

A type of microencapsulation is complex coacervation and it has been used to encapsulate small particles. It works by bringing together polymers with different charges to create a coacervate phase (11). A critical condition in complex coacervation is the pH of the reaction, as it affects the charge and solubility of the polymers (6). The optimal and critical pH values for complex coacervation depend on the type of polymers used and their concentration. Since the formation of complex coacervates is driven by electrostatic interactions, the *ζ* potential could provide information about the interactions between charged proteins and polysaccharides, as well as the formation and coacervate stability (6, 11, 12). Spray drying and freeze-drying have been well-established and widely used techniques for the microencapsulation process in the food industry (13). Of these, freeze-drying is one of the best methods for drying pigments that are sensitive to high temperatures, such as anthocyanins (13).

Coatings like gums, modified starches, whey protein, and dextrin are commonly used to protect bioactive compounds through complex coacervation (11). Peas (*Pisum sativum*) are characterized by two main globulin proteins: legumin and vicilin. The use of pea protein as an encapsulating agent in complex coacervation is notable for its ability to improve the encapsulation efficiency of bioactive compounds (12, 14, 15). Additionally, it represents attractive hydrocolloids in food industry applications due to their health benefits, such as allergen and gluten-free properties (16). Inulin, on the other hand, is a type of fructooligosaccharide prebiotic that is known for being versatile and is extracted from chicory (17, 18). Although food applications rarely use it as a coating material, Mensink et al. (19) have applied it as a cryoprotectant and lyoprotectant for the stabilization of haemagglutinin during freeze-drying. A key differentiator in this project is the incorporation of inulin as a natural biopolymer. Inulin is a well-documented fructan-type prebiotic that selectively stimulates the growth and metabolic activity of beneficial gut microbiota, particularly *Bifidobacterium* and *Lactobacillus* species. Studies have shown that inulin concentrations ranging from 2 to 10% (w/v) are effective in inducing prebiotic benefits. For example, inulin concentrations of 2 and 3% have been observed to be sufficient to stimulate the growth of probiotic cultures, while higher concentrations, such as 5%, can further enhance the viability and metabolic activity of these bacteria during processes such as fermentation or freeze-drying (20, 21).

In the context of cryoprotection, inulin has been studied for its ability to protect cells during freeze-drying processes. Although inulin shows promising results as a cryoprotectant, its protective capacity during long-term storage is limited compared to other saccharides, such as sucrose. Studies have reported cryoprotective effects with inulin at concentrations as high as 10% (w/v); however, its efficiency in maintaining stability over extended storage periods tends to be lower (20).

Anthocyanins extracted from diverse plant materials have been applied in food systems due to their health-promoting benefits. Red cabbage (*Brassica oleracea* L.) is recognized as a typical source of natural anthocyanins, offering excellent nutritional value and contributing positively to human health. It is a vegetable rich in vitamins C and K, minerals, *β*-carotene, fiber, and a high content of Cyanidin-3-glucoside (22). Additionally, studies conducted in diabetic mice have shown that cyanidin-3-glucoside, a functional group of anthocyanins, has greater effects in obesity treatments and improves hyperglycemia and insulin sensitivity (23). However, different research has shown that anthocyanins from diverse plant sources exhibit distinct pH-sensitivity profiles, which can significantly impact the films’ properties. Several studies also reported that physical, thermal, mechanical and structural properties of the films can be advantageous or disadvantageous affected by anthocyanins (3). For this reason, a study of the anthocyanin extract encapsulation by complex coacervation would provide clues for a more optimal design for the development of red cabbage extracts. Based on this, the aim of this study was the microencapsulation of a red cabbage extract containing anthocyanins through the complex coacervation of inulin, gum arabic, and pea protein as encapsulating agents and their characterization.

## 2 Materials and methods

### 2.1 Materials

Red cabbage, at full maturity and freshness, was obtained from a local market (Puebla, Mexico). The leaves were oven-dried and finely ground into powder, which was used as flour in the extraction process. Pea protein was obtained from Ingredion Mexico (Vitessence Pulse) (80% protein, dry basis). The powdered agave inulin was acquired from Hacienda Oro De Agave, and gum arabic was obtained from Ingredion Mexico. The ethanol, hydrochloric acid, potassium chloride, sodium acetate and all other reactants were of analytical grade (Sigma Aldrich, Mexico).

### 2.2 Anthocyanin-rich red cabbage extract

To resuspend 60 g of red cabbage flour, 600 mL of 1:1 ethanol and acidified water with citric acid (pH 2.5) were used (24). The solution was sonicated in a Digital sonifier (102°C, Branson) for 15 min with pulses of 20s on and 5 s off, while being kept in ice. After sonication, the solution was stirred for 15 min at 600 rpm and then centrifuged at 3,000 rpm for 15 min. The supernatant was recovered, and subjected to rotary evaporation for 5 h at 60°C. After the evaporation process, the extract was kept at 4°C for further use.

### 2.3 Differential pH method

To measure the concentration of total monomeric anthocyanins, the differential pH method described by the AOAC Official Method 2005.02 was used (25). In brief, red cabbage juice extract samples were diluted in potassium chloride buffer (pH 1.0) and sodium acetate buffer (pH 4.5). The absorbance of each solution was then measured at 520 nm and 700 nm. The free anthocyanin content was calculated using Equation 1.


(1) $$\mathrm{Free}\;\mathrm{Anthocyanins}\;\frac{\mathrm{mg}}{L}=\frac{A\;x\;MW\;x\;DF\;x\;10^{3}}{\varepsilon \;x\;1}$$

where: 𝐴 = (𝐴520 𝑛𝑚 − 𝐴700 𝑛𝑚) 𝑝𝐻1 − (𝐴520 𝑛𝑚 − 𝐴700 𝑛𝑚) 𝑝𝐻4.5; 𝐷𝐹 = 𝐷𝑖𝑙𝑢𝑡𝑖𝑜𝑛 𝐹𝑎𝑐𝑡𝑜𝑟 𝑀𝑊 (𝑀𝑜𝑙𝑒𝑐𝑢𝑙𝑎𝑟 𝑚𝑎𝑠𝑠) = 449. 2 𝑔 (for *cyanidin-3-glucoside*); 𝑚𝑜𝑙 *ε* (𝑀𝑜𝑙𝑎𝑟 𝑒𝑥𝑡𝑖𝑛𝑐𝑡𝑖𝑜𝑛 𝑐𝑜𝑒𝑓𝑓𝑖𝑐𝑖𝑒𝑛𝑡 = 26, 900) 𝐿 (for *cyanidin-3-glucoside*). The analysis 𝑚𝑜𝑙 𝑐𝑚 was performed in 0.38 cm path length cuvettes and a dilution factor of 10.

### 2.4 Inhibition of enzymatic activity of *α*-amylase

To evaluate the functional properties of red cabbage extract, inhibition percentage tests of α-amylase were conducted using different concentrations of the extract. This was done using a modified method by Ahmed et al. (26). In brief, a series of different concentrations of red cabbage extract (0.25, 0.5, 1, 2, and 5 mg/mL) was prepared. An aliquot of 250 μL of the different concentrations was placed in separate in microcentrifuge tubes of 2 mL and 250 μL of sodium phosphate buffer (negative control) 20 mM (pH 6.9) containing a solution of *α*-amylase (1.36 mg/mL) was added. The solution was incubated at room temperature for 10 min. After that, 250 μL of 1% starch solution in sodium phosphate buffer (20 mM) was added, then it was incubated for 10 min at room temperature. To stop the reaction, 500 μL of dinitrosalicylic acid (DNS) reagent 96 mM was added, and the mixture was allowed to cool to room temperature.

The reaction mixture was diluted 1:1 with ultrapure water. The absorbance at 540 nm was measured in a 96-well plate, where each well contained 100 μL of reaction mixture and 100 μL of sample according to the different extract concentrations. A similar procedure was also used for the standard drug (acarbose), which was prepared to be used as a positive control, which contained 100 μL of reaction mixture with 100 μL of acarbose (750 mM).

The inhibitory activity of *α*-amylase was calculated as the percentage of inhibition using Equation 2.


(2) $$\%\mathrm{inhibition}=\frac{\mathrm{Abs}\;\mathrm{negative}\;\mathrm{control}-\mathrm{Abs}\;\mathrm{samp}le}{\mathrm{Abs}\;\mathrm{negative}\;\mathrm{control}}*100$$

### 2.5 *ζ* potential

The ζ potential measurements were carried out in a pH range from 7 to 2.5, using a (Zetasizer Lab, Malvern Instruments, UK) equipment with three replicates. All measurements were performed at room temperature at a total biopolymer concentration of 1% w/v (in a 1:1 ratio of gum arabic:inulin) and 1% w/v pea protein. The solutions were prepared with distilled water and were left under constant agitation at 600 rpm for 24 h at room temperature. For pH adjustment, 1 and 0.1 M HCl solutions, as well as 0.1 M NaOH solutions, were used.

### 2.6 Preparations of encapsulating materials and extract

#### 2.6.1 Preparation of wall materials

A sample of 30 mL of pea protein (PP) was prepared at 1% (w/v) dissolved in distilled water. The dissolution was kept stirring overnight at 25°C. Then, it was submitted to an ultrasound bath (Bransonic 5510R-DTH, Mexico) for 30 min (12) to increase the kinetic energy of the molecules and help mix the components more efficiently. The dissolutions were realized by duplicate.

#### 2.6.2 Preparation of carbohydrate dispersions

To prepare the carbohydrate dispersions, gum arabic:inulin (GA:In) was prepared at concentrations 1 and 3% (w/v) dissolved in distilled water and kept under stirring for 1 h at room temperature (12).

#### 2.6.3 Preparation of red cabbage extract

The red cabbage extract (RCE) (30 mL) was prepared at 10 and 20% v/v with distilled water (Section 2.2). The dissolutions were agitated with a vortex for 3 min at room temperature.

### 2.7 Microencapsulation process

The microencapsulation process was carried out using a modified method described by Pintanela et al. (12). Four treatments were realized. T1: 15 mL of red cabbage extract (RCE) at 10% v/v, and 7.5-mL PP at 1% w/v were homogenized with an Ultra-Ultrax (Bio-Gen PRO200, Germany) for 3 min at 13,500 rpm. After, 7.5 mL of carbohydrate dispersions (gum arabic:Inulin) at 1% w/v were added to the solution and homogenized again under the same conditions. T2: 15 mL of RCE at 10% v/v, 7.5 mL of PP at 1 and 3% of GA. T3 and T4 were the same protocol with the unique modification of RCE (20% v/v) (Table 1). It is important to note that all concentrations used were based on the most commonly reported concentrations for complex coacervation systems.

**Table 1:** Experiment design of microencapsulation process.

Treatment	Pea protein (w/v)	Gum arabic:inulin (w/v)	Red cabbage extract (v/v)
T1	1%	1%	10%
T2	1%	3%	10%
T3	1%	1%	20%
T4	1%	3%	20%

All treatments had a 1:1:2 ratio (7.5 mL of pea protein, 7.5 mL of gums, and 15 mL of extract).

All dispersions were adjusted to the optimum pH, according to *ζ* potential (section 2.5), where the interaction between the polymers occurred to induce complex coacervation (6). To adjust to the optimum pH, HCl (1 M) was added. After adjusting the pH, the dispersions were centrifuged at 3,000 rpm at 24°C for 8 min (27). The fraction with coacervates was collected and submitted to freeze-drying. The coacervates were kept at −20°C until further use.

### 2.8 Encapsulation efficiency

To evaluate the encapsulation efficiency percentage (%EE), the encapsulated anthocyanins (EA) and the surface anthocyanin content (SA) of the microcapsules were determined using a modified method described by Laokuldilok and Kanha (10) (Equation 3).

#### 2.8.1 Encapsulated anthocyanin (EA)

A 20-mg sample was weighed and mixed with 0.2 mL of distilled water to then break the microcapsules with a vortex at room temperature for 2 min. Subsequently, 1.8 mL of ethanol was added and vortexed for 5 min. The sample was centrifuged at 6,000 rpm for 5 min, and the supernatant was collected and filtered through a 0.45 mm millipore membrane.

#### 2.8.2 Superficial anthocyanin (SA)

A 5 mg sample was weighed and mixed with 500 μL of 96% ethanol. The sample was vortexed for 30 s, then centrifuged at 6,000 rpm for 5 min. The supernatant was collected and filtered through a 0.45 mm millipore membrane.

The content of anthocyanins for the EA and SA values was determined using the differential pH method (25). To calculate the %EE, Equation 3 was used.


(3) $$\%EE=\frac{\begin{array}{l} \mathrm{Encapsulated}\;\mathrm{Anthocyanin}-\\ \mathrm{Superficial}\;\mathrm{Anthocyanin} \end{array}}{\mathrm{Encapsulated}\;\mathrm{Anthocyanin}}*100$$

### 2.9 Solubility

Solubility was determined according to the methodology proposed by Castagna et al. (28) with some modifications. Samples of 5 mg were added to 50 mL of distilled water in a precipitation flask. The solution was stirred at 100 rpm for 30 min (25°C), followed by centrifugation at 3,000 rpm for 5 min. Then, the mixture was filtered (Whatman No. 1) and a 3 mL aliquot of the filtrate was transferred into a dried and pre-weighed aluminum dish and immediately oven-dried at 105°C. Solubility was calculated gravimetrically.

### 2.10 Fourier transform infrared spectroscopy (FTIR)

The IR spectra of PP, RCE, and GA:In were obtained using an FTIR spectrophotometer (ITRracer-100, Shimadzu, Japan). To obtain the spectra in transmission mode, the samples were directly applied to a diamond ATR crystal, where each spectrum was the result of 30 scans at 4 cm^−1^. The measurements were recorded in the 4,000 to 400 cm^−1^ range. The spectra were exported and analyzed using the OriginPro^®^ (OriginLab, Northamptom, MA, United States) software.

### 2.11 Scanning electron microscopy

The morphology of the microencapsulation was observed using a using a field emission scanning electron microscope (FEI Quanta 250 FEG, Brno, Czech Republic). The samples were placed on aluminum stubs with double adhesive carbon conductive tape and coated with gold in a Quorum Q150R S (Laughton, East Sussex, England) coater for 1 min.

### 2.12 Statistical analysis

Minitab 16® software (Minitab Inc., USA) was used to perform the analysis of variance for analyzing *α*-amylase inhibition in the extract, encapsulation efficiency, particle size in the microencapsulated and solubility. Differences between average values were compared using Tukey’s test with a *p* < 0.05.

## 3 Results and discussion

### 3.1 Determination of total monomeric anthocyanin in the extract

The total monomeric anthocyanin concentration in red cabbage extract was 6.7 μg anthocyanin Eq. C3G/mg of flour. This value is higher than those reported in previous studies. For instance, Chandrasekhar et al. (29) obtained a maximum value of 0.775 μg anthocyanin Eq. C3G/mg of flour using a 1:1 ratio of ethanol and acidified water (1% HCl). Similarly, Hosseini et al. (30) reported a concentration of 0.411 μg anthocyanin Eq. C3G/mg of flour using a water/ethanol/citric acid mixture (50:48:2). These differences in anthocyanin content may be attributed to variations in extraction methods, such as solvent composition, as well as the amounts of dried samples used in each study. Nevertheless, our results demonstrate the effectiveness of the extraction protocol employed in this work.

### 3.2 *α*-Amylase inhibition

Anthocyanins have been shown to play a role in the modulation of metabolic pathways related to obesity and diabetes, sharing mechanisms with α-amylase inhibitors such as acarbose. Cyanidin-3-glucoside, the most abundant anthocyanin in red cabbage (31), has been reported to inhibit carbohydrate-digesting enzymes (32).

In this study, the red cabbage extract showed an α-amylase inhibition of 22.48% at a concentration of 0.5 mg/mL, while no inhibition was observed at lower or higher concentrations tested (Table 2).

**Table 2:** *α*-amylase inhibition.

Concentration (mg/mL)	0.25	0.50	1.00	2.00	5.00
% Inhibition	0.00 ± 0.00^A^	22.48 ± 5.66^B^	0.15 ± 0.26^A^	1.17 ± 1.64^A^	$0.00\pm 0.00^{A}$

Different letters in the same row differed statistically [*p* < 0.05].

Previous studies have reported higher inhibition levels with red cabbage extracts, such as Podsedek et al. (33), who found a 69% inhibition using an extract at 3 mg/mL, and Ji et al. (34), who reported 25% inhibition with bilberry extract anthocyanins at 4.06 ± 0.12 mg/mL. The lower inhibition observed in our study could be attributed to the lower anthocyanin concentration used, as well as potential differences in extract composition and anthocyanin stability.

While structural differences in anthocyanins, such as glycosylation patterns, may influence their inhibitory activity (33), further studies are needed to determine the specific contribution of cyanidin-3-glucoside in red cabbage extract to *α*-amylase inhibition. Additionally, more research is required to establish effective doses for potential functional food applications.

### 3.3 Interaction protein-polysaccharide through *ζ* potential

Understanding the potential ζ allows to understand how electrostatic interactions between proteins and polysaccharides with opposite charges form and maintain coacervates, contributing to their formation and stability (16). The ζ potential of pea protein and gum arabic:inulin was studied according to pH during acid titration (pH 7.0 to 2.7).

Pea protein showed a cationic nature at pH values below 3.5. This is due to the protonation of the amino groups (-NH 3+), while an anionic nature was observed at pH values above 3.5 due to the deprotonation of the carboxylate groups (COO −) (16, 35). Complex coacervates are typically formed in biopolymers below the protein’s isoelectric point. The literature reports that the isoelectric point of commercial pea protein globulins lies in the pH range of 4 to 5, which aligns with the results of this study, which found the isoelectric point at a pH of approximately 3.6 (36, 37). However, pH 3.6 was identified as the optimal condition for coacervation at a 1:1 ratio. At different ratios, other studies have reported that the optimum pH for complex coacervation may vary, depending on the specific interactions between the biopolymers involved.

**Figure 1:**
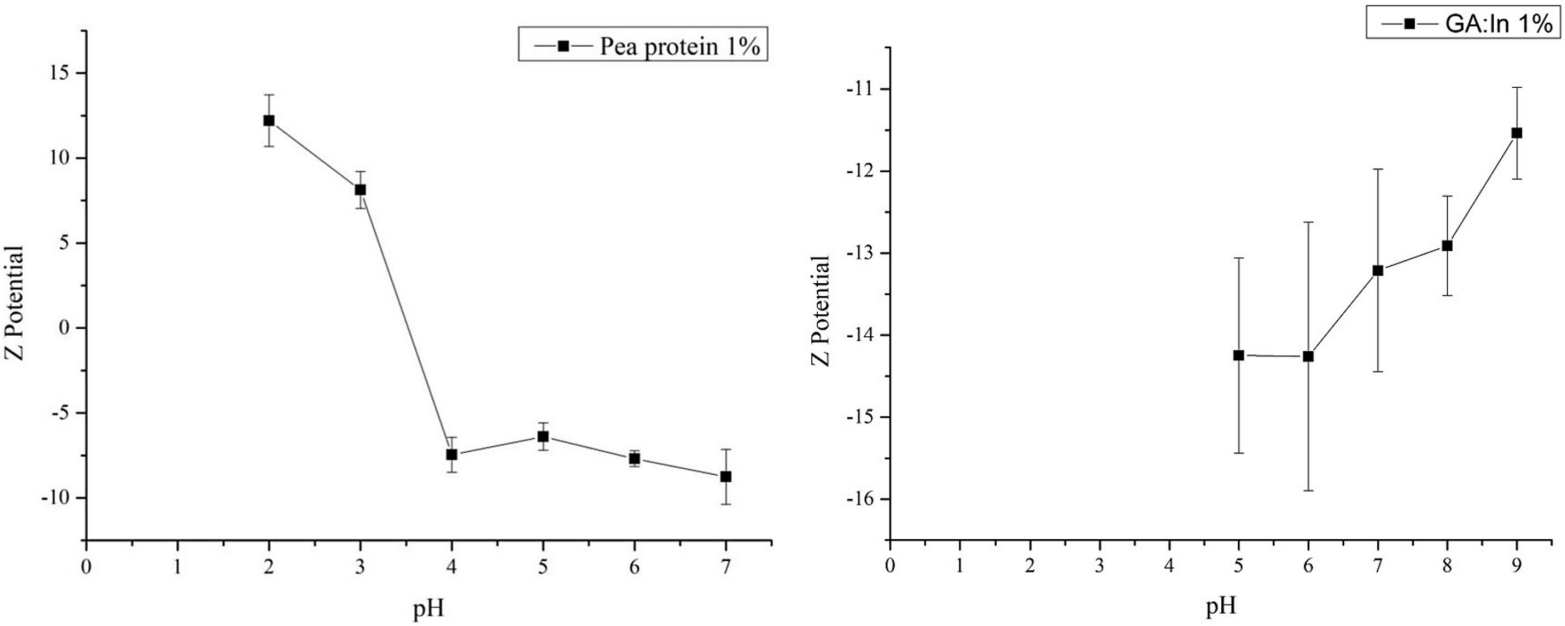
pH vs. *ζ* potential (mV) of pea protein (1%) and gum arabic (1%).

On the other hand, gum arabic:inulin consistently displayed negative potential values, with its absolute value increasing as the pH increased. Similar to what happens with anionic biopolymers, the deprotonation of carboxylate groups takes place as the pH increased. However, upon reaching higher pH values, the potential *ζ* ceased to change (38). Figure 1 displays the graph that results from the potential measurements of the protein and polysaccharides.

As previously mentioned, pH adjustment is necessary for complex coacervate formation. Researchers have reported that the optimal conditions for anionic polysaccharides lie between pH 3 and 5 (6). The above data indicated that pH 3 was the optimal pH for complex coacervation. This is because at that pH value, PP had a positive charge, and the polysaccharides had a negative charge. This implies the neutralization of charges, as the formation of coacervate occurs when the net charge of the biopolymer mixture approaches zero. Carpentier et al. (16) found that the addition of anionic polysaccharides induces the adsorption of cationic proteins through electrostatic attraction at a pH of 3 to 3.5, indicating a neutralization of the charge. Given the above, the determined pH value is consistent with reported studies (16).

### 3.4 Encapsulation efficiency

One important thing to consider with microencapsulated particles is their encapsulation efficiency, which is the ability of the envelope materials to keep the core content inside the microcapsule against bad conditions that could break it down (39). Table 3 shows the EE obtained from the different treatments applied.

**Table 3:** Encapsulation efficiency percentage and solubility of anthocyanins extracted from red cabbage extract using the mixture composed of pea protein and gum arabic:inulin as the encapsulating matrix.

Treatment	T1	T2	T3	T4
%EE	98.76 ± 2.15^A^	96.51 ± 3.36^A^	98.92 ± 1.36^A^	97.04 ± 2.30^A^
% Solubility	75.84 ± 0.15^A^	73.72 ± 0.28^B^	70.98 ± 0.04^C^	70.12 ± 0.06^D^

Different letters in the same row differed statistically [*p* < 0.05].

It is important to note that there are no significant differences among the encapsulation efficiency values across treatments, as the observed variations fall within the margin of experimental error (*p* < 0.05).

To date, no previous studies have applied the specific formulation of wall materials used in the present study, thus precluding direct comparisons regarding encapsulation efficiency. However, a study used a combination of gum arabic and inulin to microencapsulate blueberry anthocyanins through a spray-drying process, using concentrations of 5% maltodextrin DE20, 5% hi-maize, 5% inulin, and 5% gum arabic. Castagna et al. (28) obtained a percentage of encapsulation efficiency of 97.79%, which is close from the higher percentages of efficiency obtained in the present study.

### 3.5 Solubility

Solubility is a critical parameter in the food industry to ensure the efficacy of powders containing microencapsulated active ingredients (28). In this study, the solubility of the coacervates varied between 70.12 and 75.84% (Table 3), with significant differences (*p* > 0.05). They are lower than what Castagna et al. (28) found for blueberry anthocyanins that were microencapsulated with maltodextrin, hi-maize, inulin, and gum arabic as wall materials.

Complex coacervation is a technique that enables the production of microcapsules with reduced water solubility, resulting in enhanced protection of the bioactive agent by preventing its premature release and ensuring its retention within the capsules (40). However, factors like particle size and agglomeration can also influence solubility, reducing the powder’s solubility in water and influencing the water content in the capsule (41). In this context, the formation of agglomerates and the water content in the capsule could be responsible for the solubility values obtained in this study.

### 3.6 Identification of specific functional groups within the coacervates

**Figure 2:**
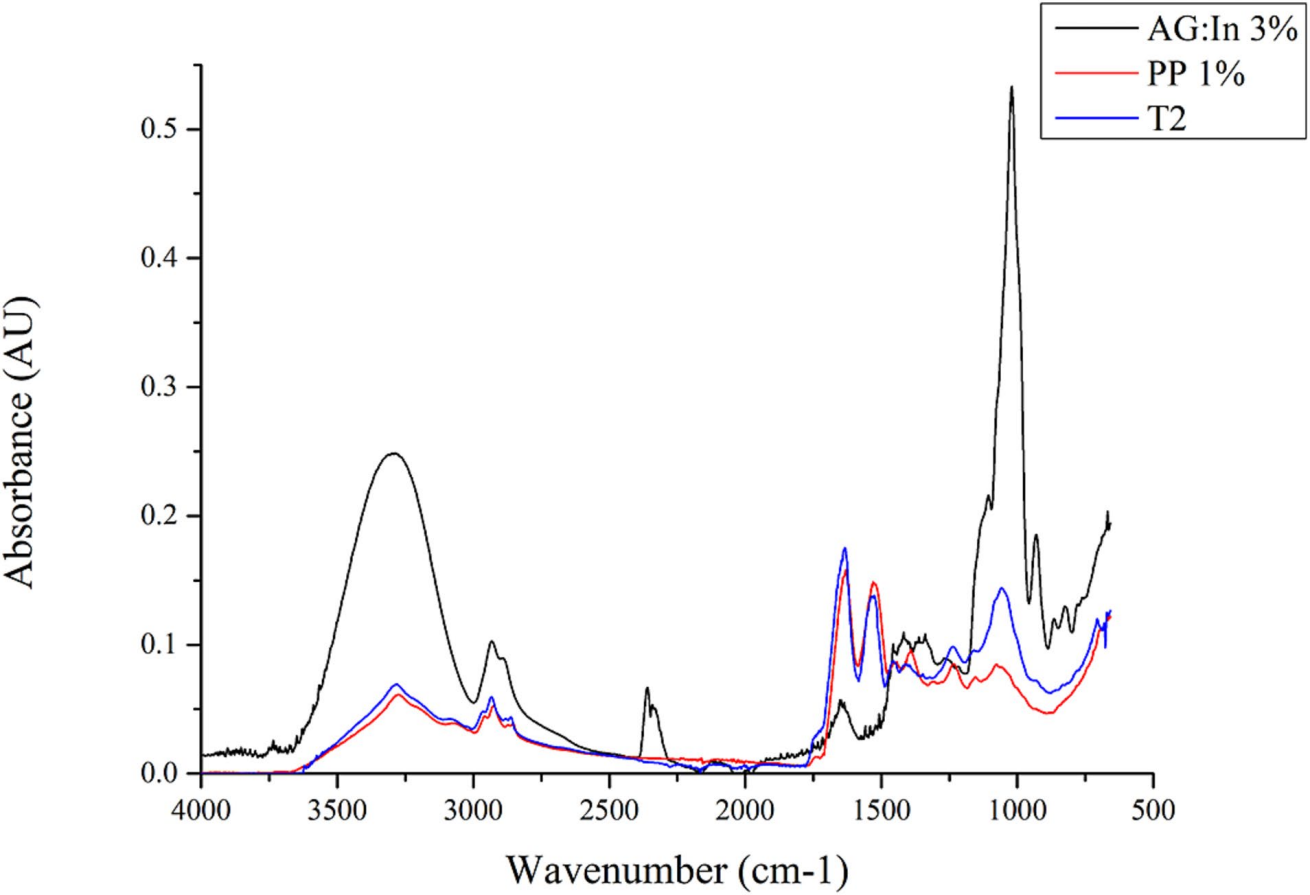
FTIR spectra of GA:In 3%, PP 1% and T2 (Pea protein 1%, GA:In 3% and red cabbage extract 20%).

**Figure 3:**
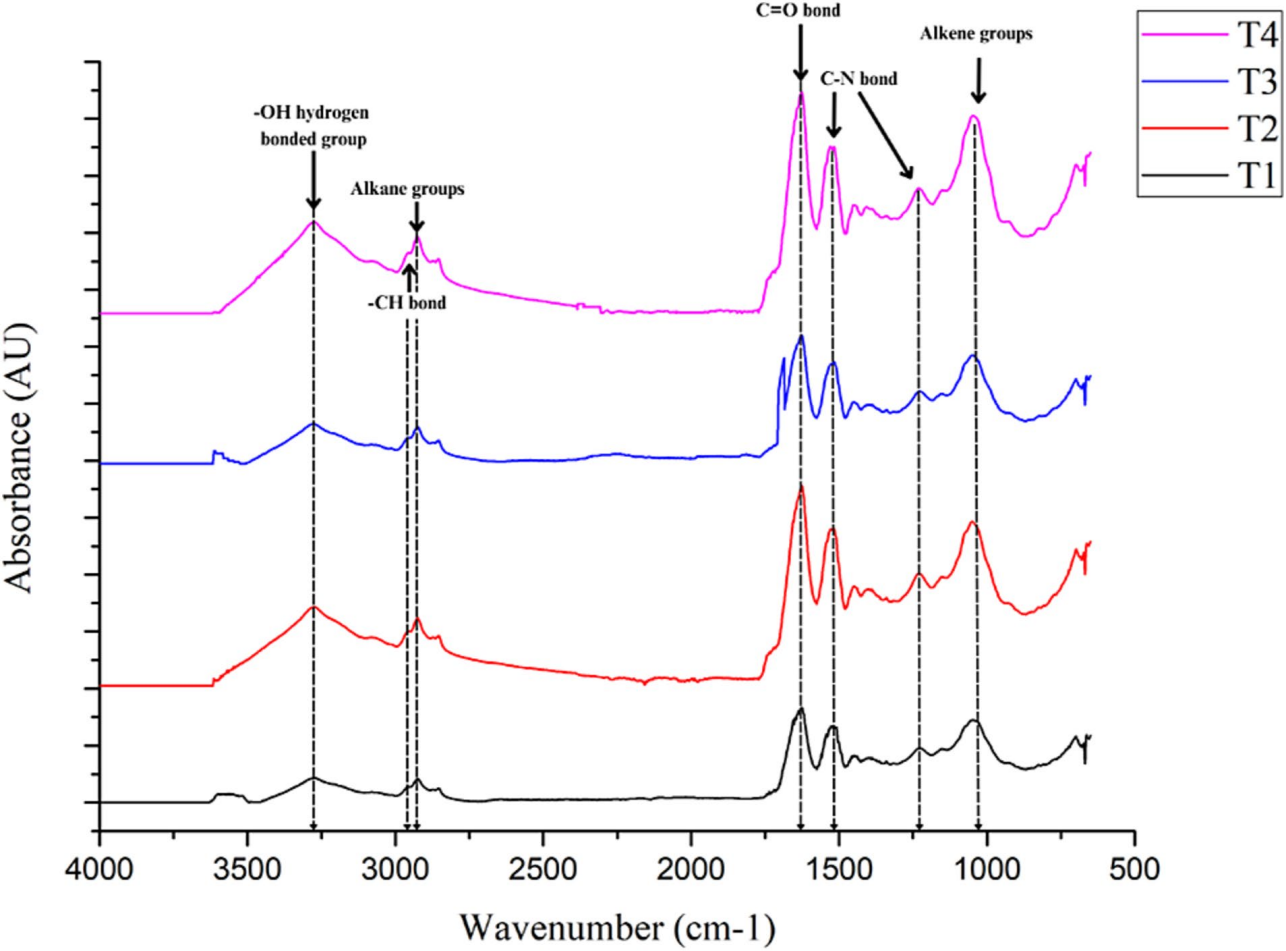
FTIR spectra of T1 (Pea protein 1%, GA:In 1%, red cabbage extract 10%); T2 (Pea protein 1%, GA:In 3%, red cabbage extract 10%); T3 (Pea protein 1%, GA:In 1%, red cabbage extract 20%) and T4 (Pea protein 1%, GA:In 3%, red cabbage extract 20%).

The formed coacervates were characterized by FTIR and SEM. FTIR analysis reveals the characteristic functional groups present in proteins and polysaccharides, as well as the interactions between them in the coacervates (16). Figure 2 shows the FTIR spectra of the raw materials, pea protein, and the homogenized red cabbage extract with protein and polysaccharide. Figure 3 shows the FTIR spectrum of the coacervates from the different treatments.

The spectrum of gum arabic:inulin 3% showed an absorption band at 3,240–3,305 cm^−1^ corresponding to -OH hydrogen bonded group, corresponding to glucosidic rings present in gum arabic. A low-intensity peak was observed between 2,850 and 1,975 cm^−1^ corresponding to alkane groups. The presence of sugars such as arabinose, galactose, and rhamnose, along with the stretching of C-H bonds, enables the observation of these bands. The bands with a low intensity at 1,640–1,629 cm^−1^, correspond to the C=C bond associated with the absorption band of the carbonyl group, which is very characteristic of carbohydrates. The strong absorption band between 1,020 and 1,040 cm^−1^ is associated with alkene groups C-H present in gum arabic and inulin (16, 42). The spectrum of pea protein exhibited a medium band at 3,280 cm^−1^ corresponding to OH-bonded groups and two low-intensity bands between 2,900 and 2,870 cm^−1^ corresponding to -CH bonds. The strong band at 1,631 cm^−1^ corresponds to C=O bending and the N-H deformation. The C-N band, which was observed at a wavenumber of 1,520 cm^−1^. Besides, the C-N stretching band was observed at 1,230 cm^−1^. These bands (1,631, 1,520, and 1,230 cm^−1^) correspond to amides I, II, and III, respectively (indicating a high content of *β*-sheet structures) (2).

Although this study did not measure the infrared spectrum of the red cabbage extract, the literature reports that the absorption spectrum of anthocyanins exhibits the following characteristics: A strong and broad absorption band at 3,276 cm^−1^ corresponds to the stretching of O-H groups in the phenolic rings, which usually absorb around 1,600 cm^−1^. There is also a weak C-H and C=C stretching band at 1,587 and 2,930 cm^−1^, respectively. An intense absorption band around 530 cm^−1^ in the region of the dactylate finger. A strong absorption band at 1,013 cm^−1^, attributable to the C=O stretching of glucoside units, as well as a broad band at 3,276 cm^−1^, which are indicative of anthocyanins substituted by glucosides.

The coacervates presented functional groups that were very similar to those in pea proteins, as well as the polysaccharides involved. This was true for both the raw materials and the microencapsulated materials. Figure 2 clearly identifies the dominant PP structure in each treatment’s spectrum. However, the coacervates infrared spectra showed bands from almost all functional groups, including proteins and polysaccharides.

It is important to mention that while T1 and T4 appeared visually similar, differences in absorbance intensity at specific wavenumbers suggest variations in molecular interactions (amide I, amide II and -OH hydrogen bonded group), likely influenced by structural rearrangements within the coacervates and hydrogen bonding between biopolymers.

### 3.7 Scanning electron microscopy

The microparticle shape in coacervates influences different characteristics, such as apparent density and retention and release mechanisms of bioactive compounds (18). In the case of microparticles obtained by freeze-drying, studies show that they usually present irregular and porous shapes (16) or spherical shapes with a rugged, fissured, and/or spongy surface, tending to form agglomerates (43). Additionally, the surface area is usually larger than that formed by emulsions through spray drying (18).

**Figure 4:**
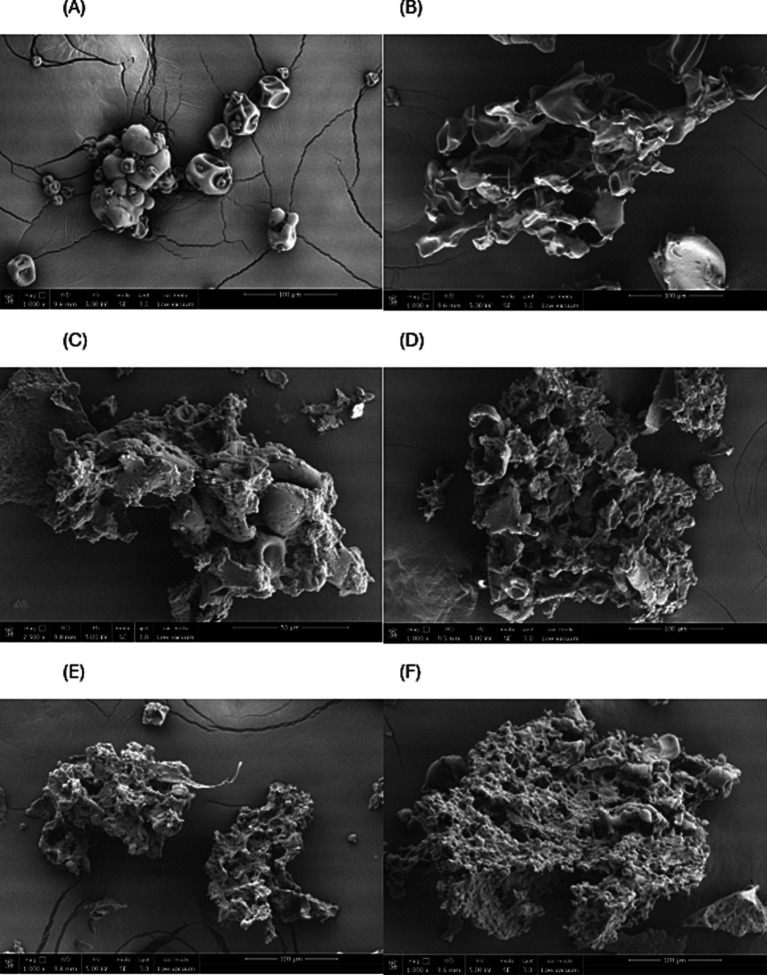
Morphologies of microencapsulated dried by freeze-drying. **(A)** Pea protein; **(B)** coacervate without red cabbage extract (Pea protein 1%, GA:In 1%, 1:1 ratio); **(C)** T1 (Pea protein 1%, GA:In 1%, red cabbage extract 10%); **(D)** T2 (Pea protein 1%, GA:In 3%, red cabbage extract 10%); **(E)** T3 (Pea protein 1%, GA:In 1%, red cabbage extract 20%) and **(F)** T4 (Pea protein 1%, GA:In 3%, red cabbage extract 20%).

In the present study, the microparticle surface morphology was observed under a FESEM microscope (Figure 4). The microencapsulated material exhibited irregular and porous shapes, as well as depressions and a rough surface probably due to the drying process (44).

Zabot et al. (18) studied the morphologies of microcapsules of berry extract using modified starch and inulin as encapsulating agents. The authors found that adding more inulin to their treatments produced microparticles with rough surfaces that resembled both round and broken or flaky particles. Carpentier et al. (16) used microencapsulation by freeze-drying pea protein with gum arabic. The microparticles showed a predominantly spherical shape but also presented a rugose surface and concavities attributed to the morphologies of pea protein and gum arabic.

## 4 Conclusion

For the first time, the combination of inulin, gum arabic, and pea protein has been reported as wall materials in complex coacervation. In this study, the encapsulation of red cabbage extract containing anthocyanins was successfully generated. This was achieved through a complex coacervation process involving inulin, gum arabic, and pea protein. The results showed high encapsulation efficiency values (up to 98.92%) and importantly, no significant impact on encapsulation efficiency was observed regardless of the polymer concentrations used. The anthocyanins exhibited functional properties, including antioxidant activity and *α*-amylase inhibition (22.48% at 0.5 mg/mL). Inulin, a prebiotic biopolymer, offered an advantage compared to other biopolymers used in complex coacervation. However, regarding its prebiotic effect, the 1% concentration used in this study is below the expected threshold to exert a significant prebiotic function. Future research will focus on increasing inulin concentration to enhance both its prebiotic and cryoprotective effects. The findings suggest that the developed encapsulates could have positive impacts on the food industry as a natural colorant. Nevertheless, further research is necessary to establish the physiological implications of red cabbage extract.

## Data Availability

The original contributions presented in the study are included in the article/supplementary material, further inquiries can be directed to the corresponding authors.
